# Symmetrization in primary tip rhinoplasty after resection of the cephalic portion of the lower-lateral cartilage

**DOI:** 10.1016/j.jpra.2023.12.008

**Published:** 2023-12-12

**Authors:** Roberto Valeriani, Edoardo Bruno, Diego Ribuffo, Maurizio Valeriani

**Affiliations:** a School of Applied Medical Surgical Sciences, University of Rome Tor Vergata, Via Montpiller 1, 00133, Rome, Italy; b Department of Surgery “P. Valdoni,” Unit of Plastic and Reconstructive Surgery, Policlinico Umberto I, Sapienza University of Rome, Via Giovanni Maria Lancisi 2, 00161, Rome, Italy; c Faculty of Medicine and Psychology, Sapienza University of Rome, Rome, Italy

**Keywords:** Cephalic trim, Lower-lateral cartilages, Face symmetry in Frankfurt's plane

## Abstract

**Background:**

The cephalic trim allows the remodeling of the alar cartilages by removing the cranial portion of the lower-lateral cartilages; this resection determines raising the tip of the nose through its rotation. The objective of this study is to demonstrate how a greater symmetry of the lower-lateral cartilages after resection of the cephalic portion is obtainable by introducing a specific additional surgical time into the procedure.

**Methods:**

Between June 2016 and December 2021, forty-six patients underwent primary rhinoplasty with the cephalic portion of the alar cartilage resection. After the cephalic trim symmetry of the nose tip was then assessed through a specific additional surgical maneuver in 23 patients (Group A), whereas in 23 patients, the evaluation of symmetry was performed according only to the surgeon's personal judgment (Group B). Preoperative and postoperative pictures were evaluated in the symmetry of the two sides of the faces considering anthropometric measurements and the judgment by ten plastic surgeons uninvolved in this study.

**Results:**

None of the patients had completely symmetric values. Objectively, the degree of asymmetry in Frankfurt's plane, considering RMLLA (midline-lateral alar margin ratio), was significantly decreased in Group A. Subjectively, more patients in Group A who were judged with asymmetrical face before rhinoplasty were evaluated with a symmetrical face after rhinoplasty than those in Group B.

**Conclusions:**

We believe that in closed rhinoplasty, the symmetry of the postoperative sides of the face is increased by performing an easily replicable intraoperative maneuver as described.

## Introduction

Nasal tip surgery has spurred the creation of more surgical techniques than any other surgical issue in the field of rhinoplasty, intending to obtain a better and seemingly natural shape and improve the balance and harmony of the face.^1^

The rhinoplasty surgeon faces the challenge of a wide variability related to both the patients’ face and nose anatomy and their requests and expected outcomes.

Furthermore, the anatomical elements frequently prevent the surgeon from applying a single technique to enhance the nose tip.^2^

Aesthetic rhinoplasty has greatly evolved over time and extremely aggressive techniques that included resection, transection, morselization, and incisions have been replaced by techniques that are reversible and significantly less damaging.

Cephalic trim is often the only technique implemented in noses with bulbous or boxy domes; with the aim to reshape the alar cartilages by removing the cranial component from the lower-lateral cartilages.^2^ The resection of the cephalic portion of the alar cartilages allows the elevation of the nose tip through its rotation.^3^

The cephalic trim only allows the rotation of the tip and not greater projection, which can only be achieved with the aid of a cartilage graft.

Among the fundamental issues when performing a cephalic resection of the lateral cartilages, especially in closed rhinoplasty, there is the accurate symmetrization of lower-lateral cartilages following the excision of the cephalic portion.

We believe that an objective method of intraoperative evaluation should be adopted to achieve the aforementioned symmetry in the replacement of the solely aesthetic judgment and the experience of rhinoplasty surgeons.

The aim of this article is to describe an operative maneuver that is useful for the evaluation of the best possible symmetry after the remodeling of lower-lateral cartilages in closed rhinoplasty through an transcartilaginous incision, and our clinical experience.

## Materials and methods

### Surgical technique

**Figure 1:**
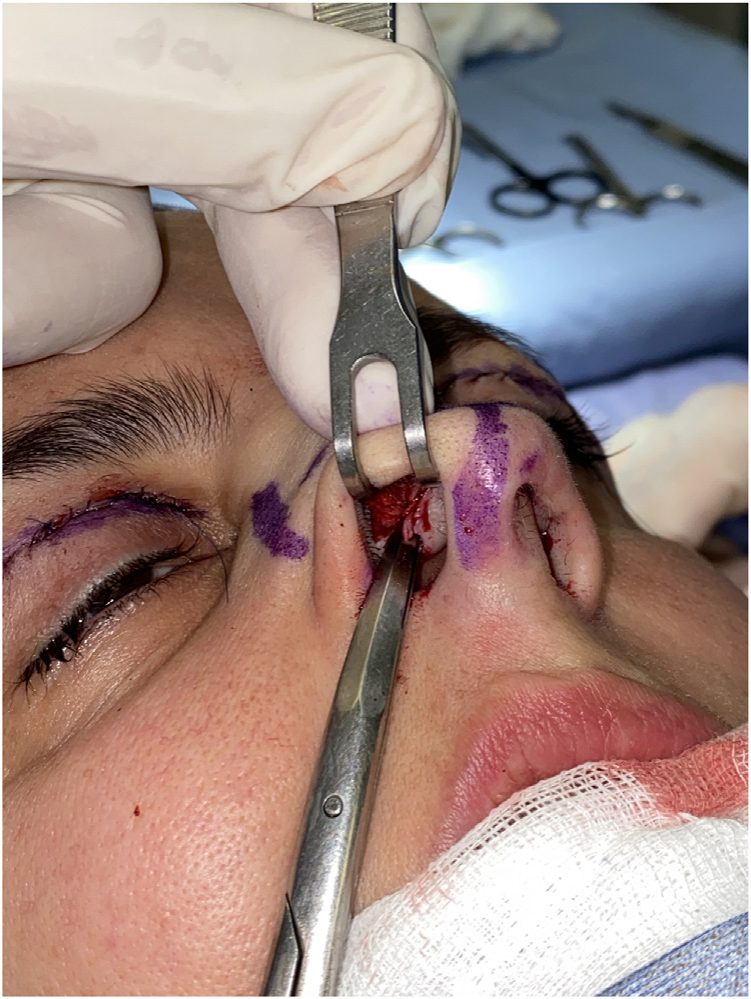
Separation of the perichondrium of lower-lateral cartilages from the vestibular mucosa after transcartilaginous incision.

Alar cartilage is exposed by flipping the nostril rim with a hook. An transcartilaginous incision is performed parallel to the nostril rim at the meeting point between the lower two-thirds and the upper third of the alar cartilage's height using a 15-blade. Vestibular skin is grabbed with a mosquito forceps so that the cephalic portion of the lower-lateral cartilage is exposed. Complete separation of perichondrium from the mucosa is performed, followed by the removal of the cephalic portion of the lower alar cartilage (Figure 1). The appearance of the nose tip is modified, while the continuity of the arch given by the lower alar cartilages is preserved.

The preservation of 6 mm of cartilage from the nostrils rim is required so as not to excessively weaken the alar cartilages. This is considerable when tip suture techniques are performed (interdomal or transdomal) exploiting the lower-lateral cartilages to improve dome shape and increase rigidity; retraction during inspiration and external valve insufficiency can result from the excessive weakening mentioned above.^2^

**Figure 2:**
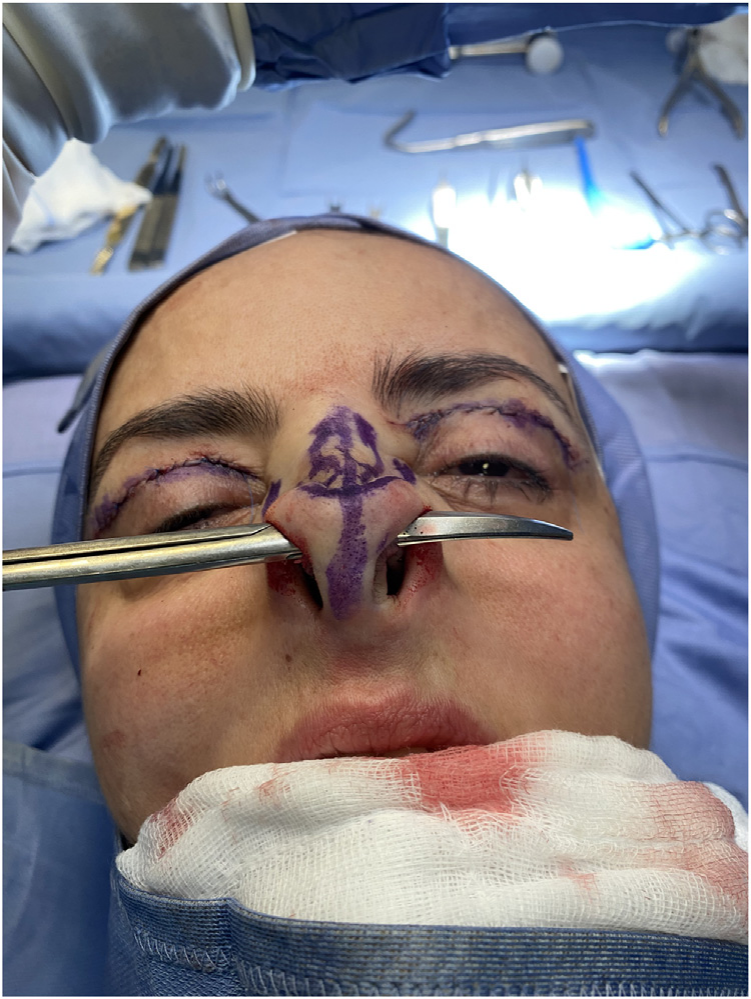
Straight Metzenbaum scissors are inserted between the transcartilaginous incisions, passing them over the residual alar cartilages in order to accurately evaluate the symmetry of the cartilaginous arches resulting from the removal of their cephalic portion.

We believe that after the removal of the cephalic portion of the lower alar cartilages, an assessment of the symmetry of the cartilaginous arches resulting from such resection should be carried out before further surgical steps. A symmetry assessment must be conducted by placing straight Metzenbaum scissors in the site of transcartilaginous incisions and passing them over the residual alar cartilages (Figure 2). This maneuver allows a clear and reproducible assessment of the symmetry in the height of the lateral branches of the two lower alar cartilages, and the necessity for further trimming of cartilage from either cartilage can be assessed.

**Figure 3:**
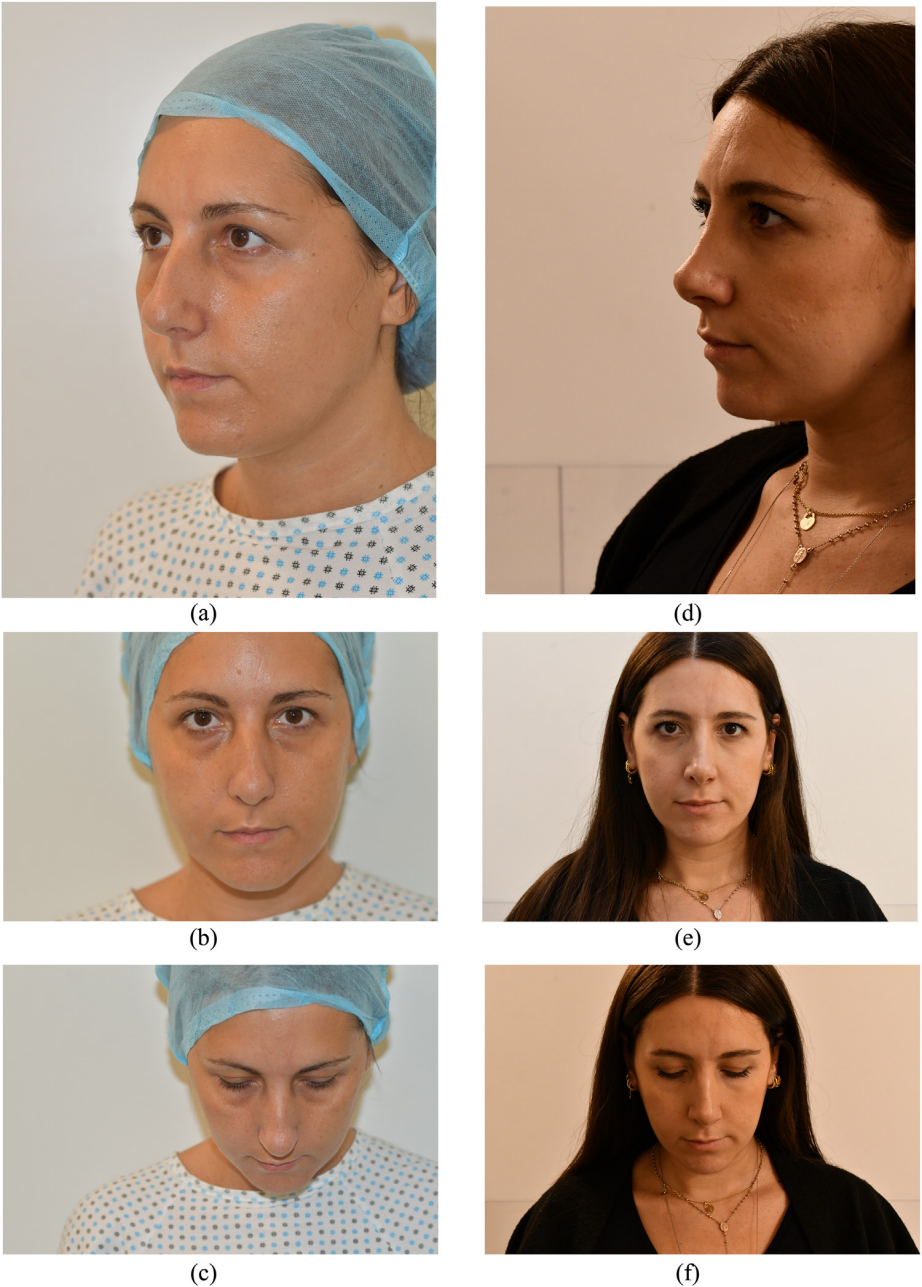
Preoperative (**a-c**) and postoperative (**d-f**) views.

Furthermore, the above-mentioned maneuver allows the separation of the subcutaneous tissue from the perichondrium and the consequent skin of the nasal pyramid region readjustment to the newly shaped cartilaginous structure. Merocel epistaxis packing (Medtronic Xomed, Jacksonville, FL) was used to stent the nasal airway for 2 days postoperatively (Figure 3a-f).

### Clinical experience

Forty-six patients have undergone primary rhinoplasty with the resection of the cephalic portion of the lower-lateral cartilages between June 2017 and December 2022. The patients included in the study were 9 males and 37 females with an average age of 38 (age range 22 to 51 years). All patients were North-European.

The following exclusion criteria were applied: less than 18 years of age and more than 55 years, history of face or nose surgery, and presence of craniofacial abnormalities.

All patients signed informed consent for the procedure. All the rhinoplasties were performed by the author of this article (Valeriani M. MD) always implementing the same closed approach.

Cephalic trim was performed in all patients included in the study, and the symmetry of the nose tip was assessed through the designated intraoperative maneuver in 23 patients (Group A), whereas in the remaining 23 patients, the nose tip symmetry was evaluated intraoperatively according to the surgeon's personal judgment (Group B). The average follow-up period for each patient was 8 months (range: 6-12 months)

Preoperative and postoperative frontal view photographs of 46 patients taken before and 6 months after the aesthetic rhinoplasty procedure in the Frankfurt position were analyzed considering the symmetry of the face. All pictures were taken by the same photographer specialized in facial plastic surgery photography and special attention to avoiding the subject's head rotations was paid. Additionally, each picture was inspected to search for any minimum axis rotation and patients whose pictures were found to have the said rotation were excluded from further analysis (n=3). Unfortunately, we were unable to eliminate a certain degree of error because of the imperceptible axis rotation.

**Figure 4:**
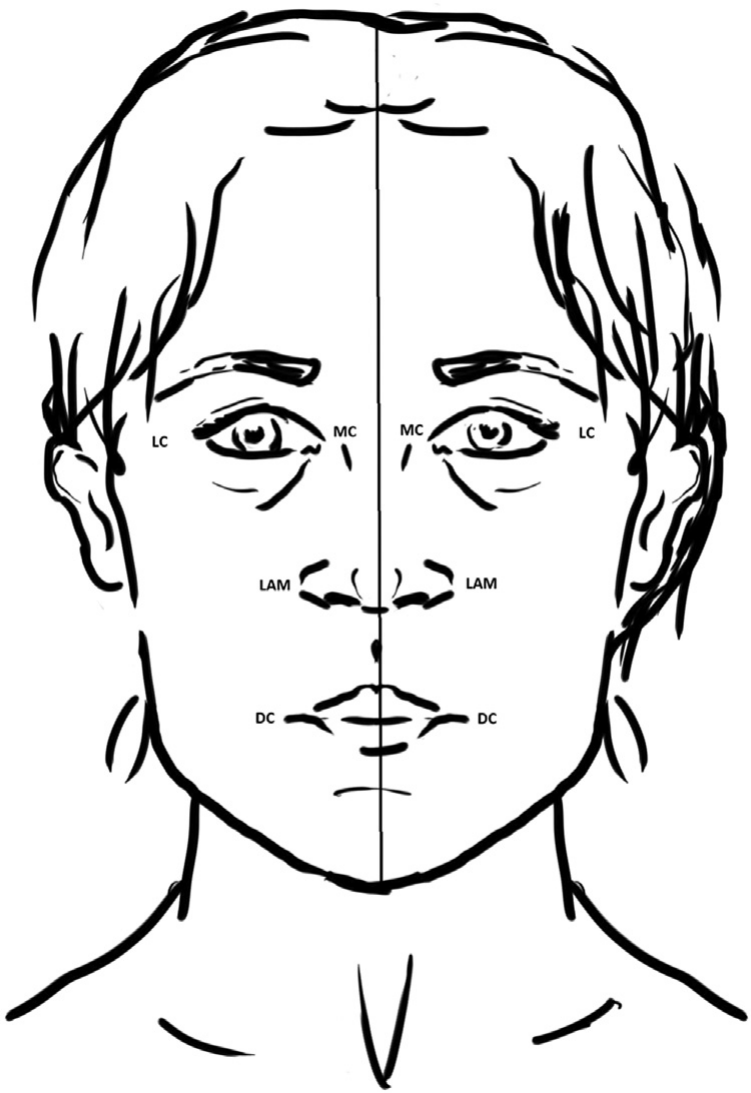
Facial anatomical landmarks for facial anthropometric measurements.

Anthropometric measurements were evaluated considering the main soft tissue landmark of the face (Figure 4)^4^, ^5^, ^6^ and using Adobe Photoshop CS3 software. Particularly, for the sake of symmetry assessment, the following elements were taken into consideration: the sagittal line crossed the central point above the bridge of the nose, the central portion of the Cupid's bow, and the most caudal point of the chin. Distances from the sagittal line were then measured for the following points: lateral alar margin, medial canthus, lateral canthus, tragus, and oral commissure.

Anthropometric measurements were taken in pixels. A ratio was calculated by dividing the measurements from one side of the face by the measurements of the other side.

The measurements of each side of the face taken into consideration were midline-lateral alar margin (MLLA), midline-lateral canthus, midline-medial canthus, midline-oral commissure, and midline-tragus.

The percentage of asymmetry was calculated with the following formula: (Ratio-1) × 100. Asymmetry degree values were grouped into >2% and >5%. Each measurement was deemed as symmetrical if the ratio was equal to 1.

The side view asymmetry was not considered in this study, as the ratio was calculated always on the frontal plane.

A statistical analysis was carried out using SPSS software. t-test was used to compare the preoperative and postoperative measurements. A P-value of 0.05 was considered statistically significant.

Furthermore, preoperative and postoperative pictures were evaluated by ten plastic surgeons uninvolved in the study and unaware of its premise. The pictures were shown sequentially to each of the observers onto a PowerPoint presentation (Microsoft Corp, Redmond, Washington); specifically, pictures were shown for 3 seconds, then 3 more seconds were given to the observer to express their opinion about the symmetry or asymmetry of the face in the pictures.

Concerning “perceptual contamination” because of the observers having to judge both preoperative and postoperative pictures of the same patient, we showed all of the forty-six preoperative pictures and the forty-six postoperative pictures in separate sessions in 4 months.

We deemed symmetrical those faces considered as such by more than 50% of the observers.^7^

## Results

In Group A patients, where the intraoperative symmetry assessment maneuver was performed, the ratio of the MLLA distance increased from 89.6% (±4.5%) before surgery to 95.8% (±5.5%) after surgery (P<0.001 paired t-test). No significant changes were observed in the other measurements (P>0.10) (Table 1).

**Table 1:** Degree of Facial Symmetry Between Group A and Group B Before and After Rhinoplasty.

Variable	R_MLTr_	R_MLLC_	R_MLMC_	R_MLLAM_	R_MLOC_
Group A (n=23) Before Rhinoplasty After Rhinoplasty Group B (n=23) Before Rhinoplasty After Rhinoplasty	95.5 (3.2) 95.8 (3.6) 95.1 (4.4) 95.4 (4.2)	92.8 (3.8) 92.6 (3.5) 93.4 (4.5) 93.6 (4.4)	91.2 (4.9) 91.4 (4.5) 91.1 (4.4) 91.5 (5.1)	89.6 (4.5) 95.8 (5.5) 89.5 (4.7) 92.7 (5.1)	91.4 (4.8) 90.8 (4.4) 92.8 (4.1) 93.7 (4.5)

Abbreviations: ML-LAM, midline to lateral alar margin; ML-LC, midline to lateral canthus; ML-MC, midline to medial canthus; ML-OC, midline to oral commissure; ML-Tr, midline to tragus; R, ratio.

**Figure 5:**
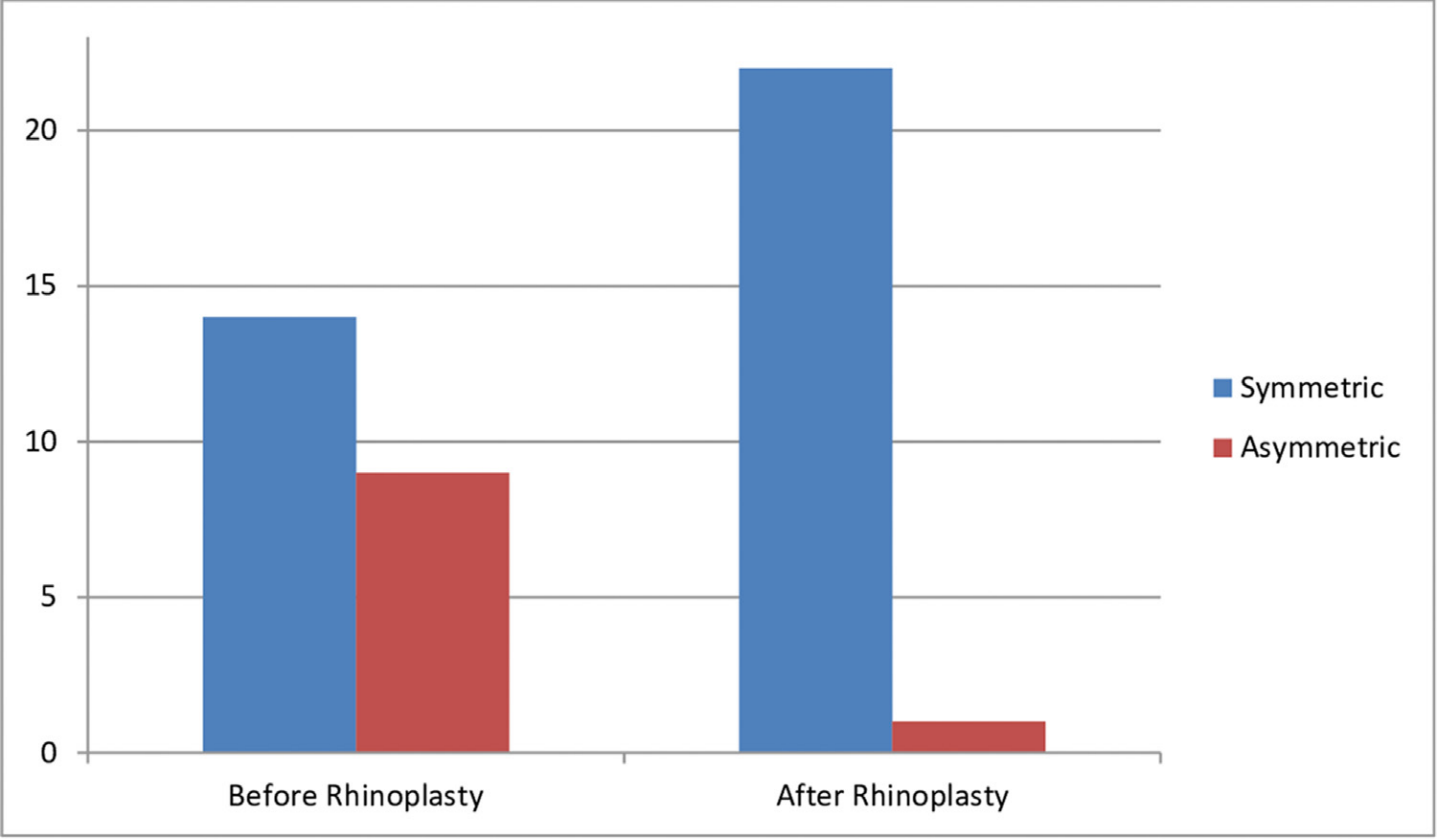
Subjective assessment of facial symmetry in Group A: patients (n=23) before and after rhinoplasty surgery.

A total of 14 patients belonging to Group A were considered with a symmetrical face before rhinoplasty, based on their preoperative pictures; this value increased to 22 patients after surgery, based on their postoperative pictures (P < 0.001, χ2). Of the 9 patients whose face was evaluated as asymmetrical before surgery, 8 were then considered symmetrical after surgery, whereas 1 was confirmed as asymmetrical even postoperatively (Figure 5).

**Figure 6:**
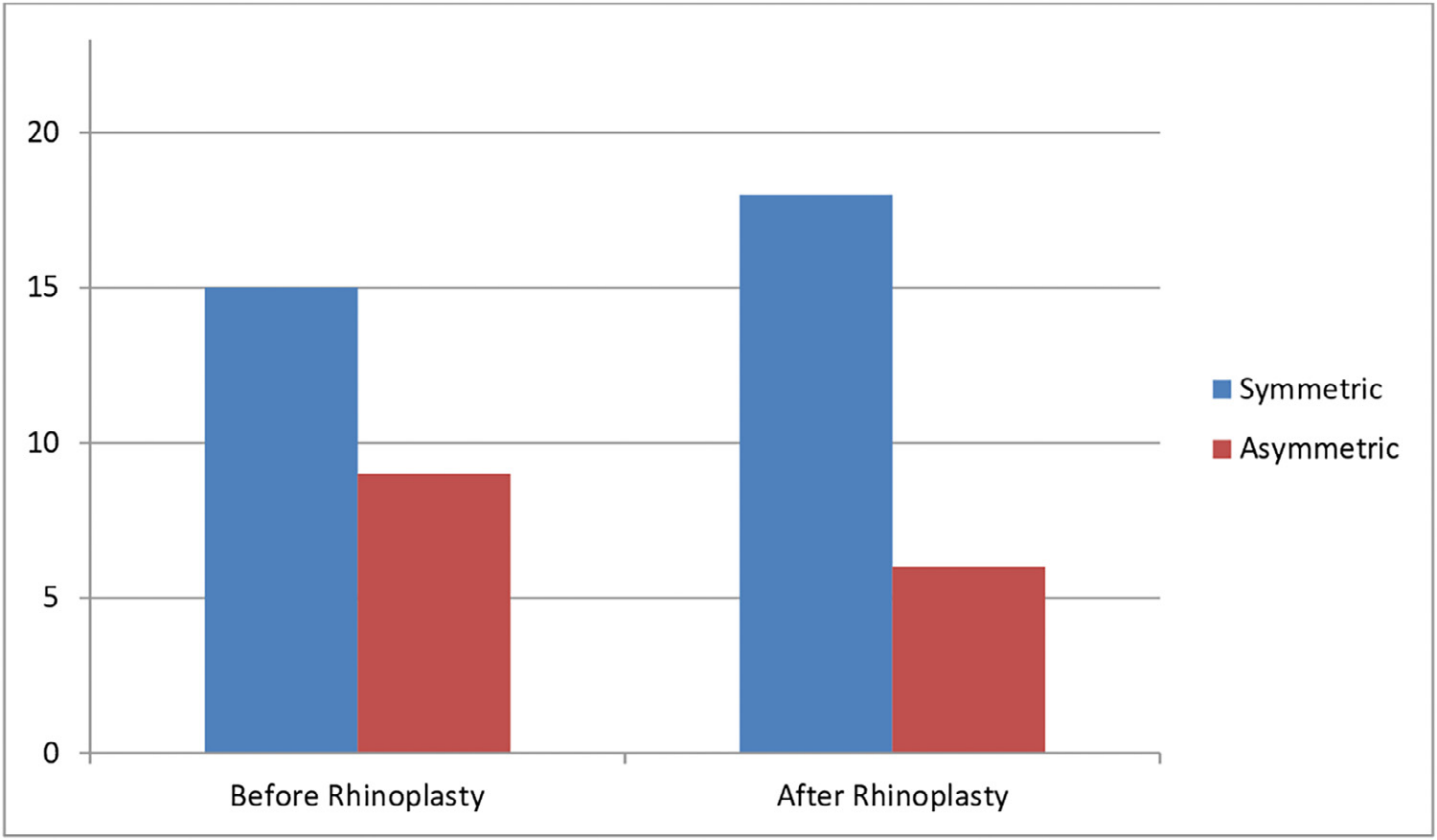
Subjective assessment of facial symmetry in Group B: patients (n=23) before and after rhinoplasty surgery.

In Group B, in which nose tip symmetry was evaluated intraoperatively based only on the surgeon's personal judgment, the increase in the mean ratio of MLLA distances was significantly smaller compared to Group A, from 89.7% (±4.7%) before rhinoplasty to 92.5% (±5.1%) after rhinoplasty (P<0.001 paired t-test). This group, as well, saw no significant modifications of the other anthropometric measurements after surgery (P<0.10). Before surgery, the faces of 15 patients were judged as symmetrical; this value increased to 18 after rhinoplasty. The faces of 9 patients of Group B were deemed asymmetrical during evaluation before surgery, 3 of these were then considered symmetrical at the evaluation of the photos taken after rhinoplasty (Figure 6).

The following postoperative complications were encountered: mucoperichondrial lacerations (2 patients, 4.34%), postoperative bleeding (1 patient, 2.17%), and surgical wound dehiscence (1 patient, 2.17%).

## Discussion and conclusions

Symmetry is achieved when one side of the face is identical to the other side. Symmetry is given by the overall balance of all the structures of the face. Although each facial feature plays a role in determining symmetry, some parts contribute more than others; in fact, the nose and chin may affect symmetry more heavily because of their central position on the face.^8^, ^9^

Achieving symmetry should be a goal in every cosmetic procedure of nasal outline remodeling.^10^

The authors consider cephalic trim as a fundamental instrument for surgeons who seek to enhance nose tip symmetry via transcartilaginous incision reshaping LLC. The authors also believe that the shape of the alar cartilages has a significant impact on the outcome of nose tip reshaping rhinoplasty.

Moreover, we believe that nose tip modification should be performed before any surgery of the nose hump, as it would allow a prior evaluation of any effect that such surgery would have on the nose tip projection resulting from LLC modifications. The cephalic trim excisional technique may reduce nose tip projection, and thus, it is often performed before the positioning of a columellar strut.^2^

Contrariwise, the Ozmen flap technique is performed during an open rhinoplasty and requires that the cephalic portion of the alar cartilage is not removed, but rather incised and pocketed between the residual cartilaginous portion and the mucosa. This technique, therefore, allows a repositioning of the cephalic portion of the alar cartilage, which can provide a combined spreader and/or splay graft effect for primary rhinoplasty with dorsal hump reduction.^11^

The refinement of a wide nose tip is achievable through the removal of the cephalic portion of lower-lateral cartilages. This procedure is commonly used to correct any nose tip deformity resulting from excessive horizontal extension, thus allowing alar cartilages to curve without collapsing.^12^

In this study, we evaluated and documented the presence of facial asymmetry before and after rhinoplasty in two groups of patients, based both on anthropometric measurements and the personal symmetry or asymmetry perception of observers not included in the study.

The use of absolute numerical values in anthropometric measurements for the face might be confusing; for this reason, the use of proportions was recommended during these evaluations.^13^ In our study, we took into consideration the ratio between the anthropometric measurements of each side of the face. Specifically, the ratio was calculated by dividing measurements from the most asymmetrical side by those from the less asymmetrical side. The face was then considered symmetrical if the above-mentioned ratio was equal to 1.

The degree of asymmetry in Frankfurt's plane, in relation to MLLA, was significantly decreased in the patients of the group where the intraoperative nose tip symmetry evaluation maneuver was performed. Symmetry improved compared to preoperative conditions, although to a lesser degree, even in cases where the intraoperative symmetry assessment maneuver was not performed.

Symmetry improvement obtained after rhinoplasty with intraoperative evaluation based on soft tissue landmarks resulted as statistically significant (P<0.05).

In the subjective evaluation of facial asymmetry, 9 of the 23 patients from Group A were considered asymmetrical (39.1%) before rhinoplasty; 8 of those patients were considered symmetrical after rhinoplasty, thus increasing the percentage of patients with facial symmetry by 34.8%. Group B also saw an increase in patients considered symmetrical comparing pre- and postoperative pictures, although this increase was only by 13%.

Researchers, however, were not able to find every asymmetry based solely on their subjective evaluation.

In conclusion, our study has demonstrated how, in closed rhinoplasty, we designed an intraoperative maneuver that is easily replicable and allows us to evaluate the symmetry between the lateral branches of the LLCs resulting from the removal of their cephalic portion, thus allowing us to achieve a better symmetry between face sides after surgery.

## CRediT authorship contribution statement

**Roberto Valeriani:** Conceptualization, Methodology. **Edoardo Bruno:** Data curation, Writing – original draft. **Diego Ribuffo:** Supervision. **Maurizio Valeriani:** Writing – review & editing.
